# Nasopharyngeal Carcinoma Treated With Photoimmunotherapy

**DOI:** 10.7759/cureus.49315

**Published:** 2023-11-23

**Authors:** Hiroshi Idogawa, Takeshi Shinozaki, Wataru Okano, Kazuto Matsuura, Ryuichi Hayashi

**Affiliations:** 1 Otolaryngology-Head & Neck Surgery, Hokkaido University, Sapporo, JPN; 2 Head and Neck Surgery, National Cancer Center Hospital East Japan, Kashiwa, JPN

**Keywords:** tumor-targeted monoclonal antibody, nasopharyngeal cancer (npc), head and neck cancer, local recurrence, photoimmunotherapy

## Abstract

Photoimmunotherapy is a new treatment modality in which a tumor-targeting monoclonal antibody is combined with a photoactivated dye and a laser is applied to destroy tumor cells. In Japan, insurance reimbursement for this treatment started in January 2021 for unresectable locally advanced or locally recurrent head and neck cancer. We used photoimmunotherapy to treat two patients with recurrent nasopharyngeal squamous cell carcinoma (NPSCC). The first patient was diagnosed with NPSCC (T1N0M0) and treated with definitive radiotherapy, leading complete response. A local recurrence was observed and treated with photoimmunotherapy. Seven months have passed, complete response is archived. The second patient was diagnosed with NPSCC (cT2N1M1). Multimodal therapy led to a complete response for all lesions. A local recurrent lesion appeared, and photoimmunotherapy has been repeatedly performed. The lesion was controlled as a stable disease for about one year. Photoimmunotherapy could be an effective treatment for local recurrence of NPSCC after radiotherapy.

## Introduction

Photoimmunotherapy is a new cancer treatment that specifically destroys tumor cells by illuminating them with 690-nm red light after the administration of cetuximab sarotalocan sodium, which is a photosensitive complex of the chimeric anti-human epidermal growth factor receptor monoclonal antibody cetuximab and a photoactivated dye (IRDye700DX IR700; Rakuten Medical, Tokyo, Japan) [1,2]. Approximately 90% of head and neck cancers have cell surface expression of EGFR. In a phase 1/2a multicenter, open-label study of photoimmunotherapy for head and neck cancer, which did not include nasopharyngeal carcinoma (NPC), the overall response rate was 43.3% for treatment efficacy. Median overall survival was 9.30 months, and median progression-free survival (PFS) was 5.16 months [3].

In Japan, the drug (Alluminox^TM^, Rakuten Medical) and devices for photoimmunotherapy were approved in September 2020, and the procedure became covered by national health insurance for clinical use to treat unresectable locally advanced or locally recurrent head and neck cancer in January 2021 [4]. Photoimmunotherapy has only been in clinical application for a short period of time, and the actual clinical outcome of photoimmunotherapy remains unclear.

Here, we describe our experience in two cases of locally recurrent NPC successfully treated by photoimmunotherapy.

## Case presentation

Case presentation 1

**Figure 1:**
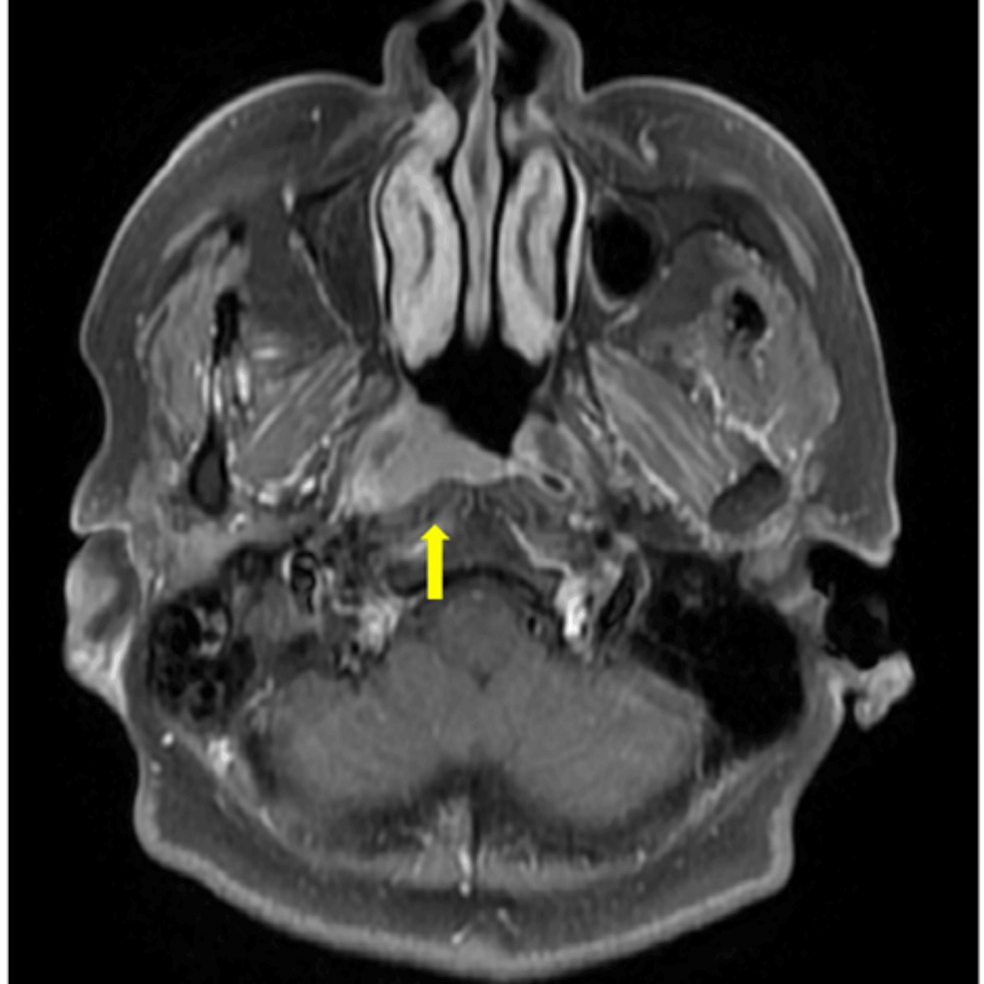
Magnetic resonance imaging before the first treatment The tumor (arrow) was diagnosed as cT1.

**Figure 2:**
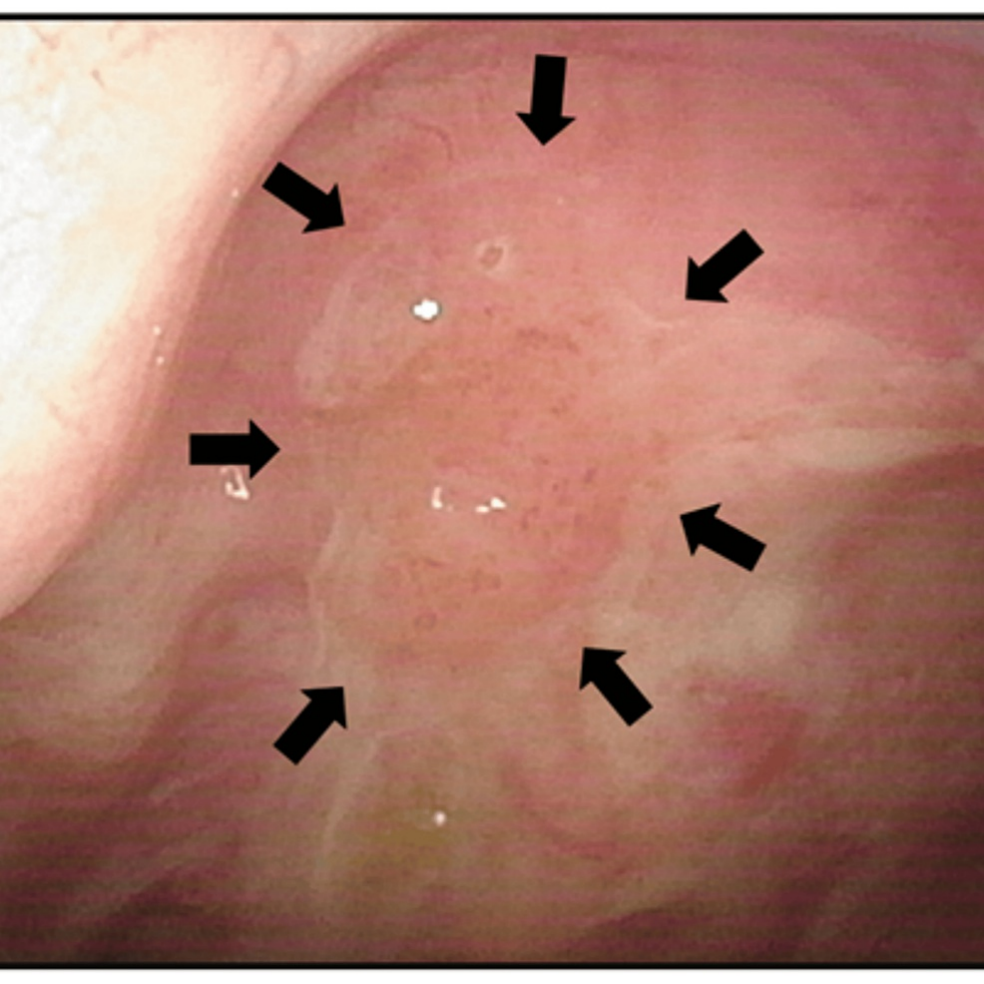
Fiberscopic finding of local recurrence six months after completion of radiotherapy The recurrent lesion (rT1) (arrows) was confined near the right edge of the posterior wall.

The patient was a 63-year-old woman. She was initially diagnosed as having nasopharyngeal squamous cell carcinoma (cT1N0M0, UICC 8th edition) that was negative for Epstein-Barr virus-encoded small RNA by in situ hybridization (Figure 1). Definitive radiotherapy (70Gr/35fr) was performed, and a complete response was achieved. However, six months after the completion of radiotherapy, a local recurrence with irregular mucosa was observed with a video endoscope, and squamous cell carcinoma was diagnosed histologically (Figure 2). Subsequently, she was referred to our hospital. The relapse was confined in the posterior wall of the nasopharynx and was too small to detect by magnetic resonance imaging (MRI). It was diagnosed as rT1. We proposed photoimmunotherapy, and the patient opted for this treatment.

Cetuximab sarotalocan sodium was administrated intravenously at the standard dose of 640 mg/m^2^. No adverse effect occurred during the intravenous administration, after which the patient stayed in a darkened room with illumination of less than 120 lx to avoid photosensitivity.

**Figure 3:**
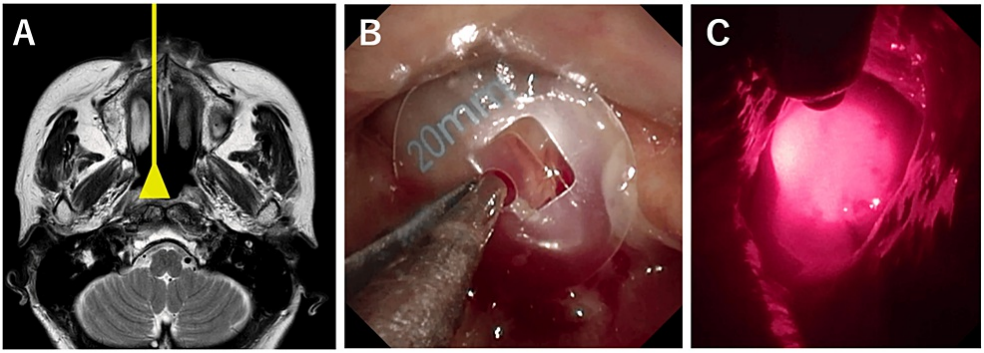
Findings of the first photoimmunotherapy in Case 1 A: Interpretation diagram of laser irradiation to the nasopharynx. B: The laser irradiation diameter was set to 20 mm to completely cover the tumor. C: The image during illumination. A frontal diffuser was placed 34 mm from the posterior wall through the right nasal cavity.

The day after cetuximab sarotalocan sodium administration, we performed laser irradiation using a photodynamic therapy semiconductor laser (BioBrade^Ⓡ^ laser; Rakuten Medical) with photodynamic therapy semiconductor laser probes (BioBrade^Ⓡ^ frontal diffuser, Rakuten Medical). After induction of general anesthesia, gauze soaked in 0.02% epinephrine was placed in the bilateral nasal cavities to shrink the mucosa. A long Killian nasal speculum (80 mm) (Nagashima Medical Instruments Co., Ltd., Japan), and a rigid endoscope were used to maintain the operating field via the right nasal cavity. After confirming that the tumor could be covered with a clear scale of 20 mm in diameter, a frontal diffuser was placed 34 mm from the posterior wall and it illuminated the whole tumor. Laser irradiation was then performed for 5 minutes 33 seconds (Figure 3). The patient resumed oral intake on postoperative Day 1 and was discharged on Day 4. After laser irradiation, a crust formed on the surface of the tumor. Five weeks postoperatively, the tumor had disappeared completely.

Six months later, a recurrence localized to the posterior wall was observed. No deep invasion was evident on MRI. Repeat photoimmunotherapy was performed using the same procedure as before, achieving a complete response.

Three months later, a locally recurrent lesion was detected from the posterior to the right lateral wall just before the pharyngeal orifice of the Eustachian tube. Repeat photoimmunotherapy was performed again. We attempted to irradiate the laser with a 20 mm diameter area but could not do this because the frontal diffuser collided with the posterior edge of the nasal septum.

**Figure 4:**
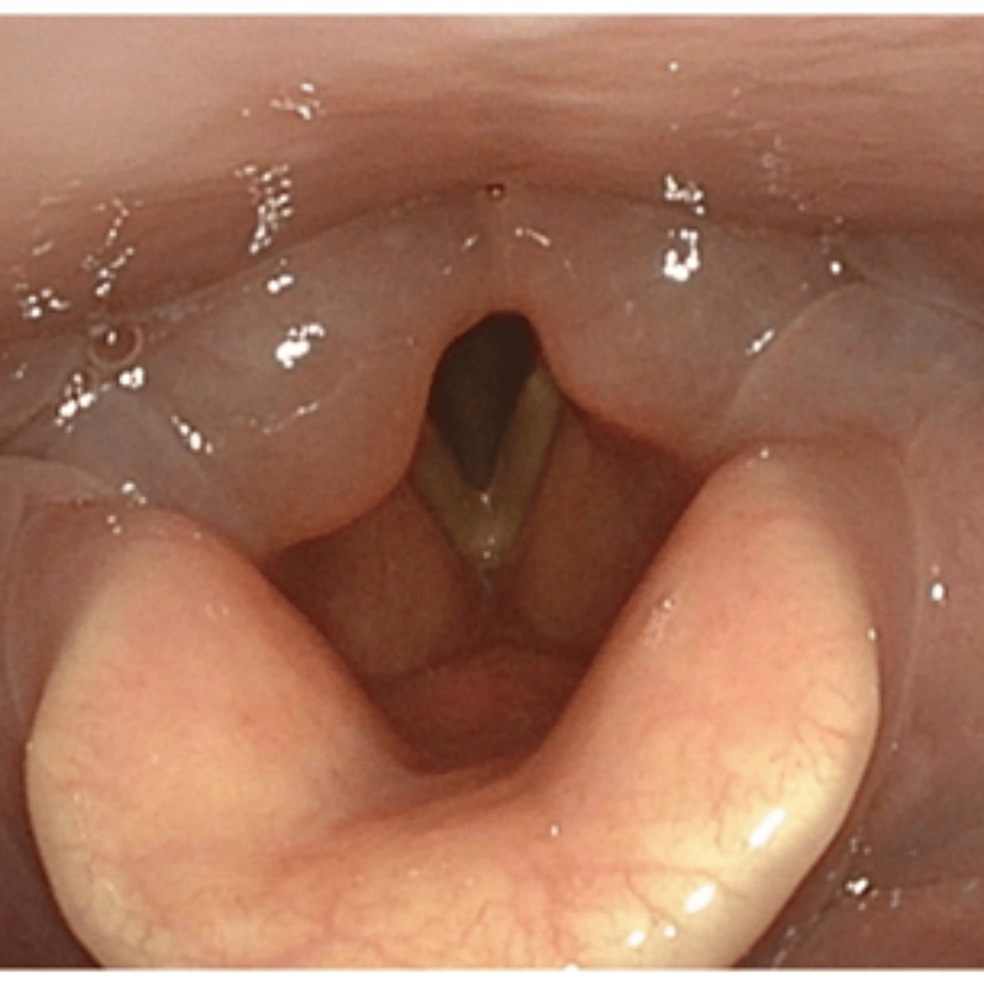
Endoscopic findings of laryngeal edema after the third photoimmunotherapy Laryngeal edema and hoarseness appeared on postoperative Day 1 after the third photoimmunotherapy and resolved the following day after treatment with hydrocortisone sodium succinate 300 mg.

A frontal diffuser was placed 30 mm from the posterior wall to illuminate a diameter of 17 mm. A 100-mm needle catheter was used to puncture dorsal to the pharyngeal orifice of the Eustachian tube, and a 20-mm cylindrical diffuser was inserted into the catheter and irradiated. Both procedures were performed through the right nasal cavity. Laryngeal edema (especially bilateral arytenoids) and hoarseness appeared the following day. Hydrocortisone sodium succinate 300 mg was administrated intravenously, and the edema resolved by the next day (Figure 4). The tumor showed further shrinkage and a fourth treatment was given one month later. Treatment efficacy achieved complete response and the patient has been without recurrence for seven months up to the present.

Case presentation 2

**Figure 5:**
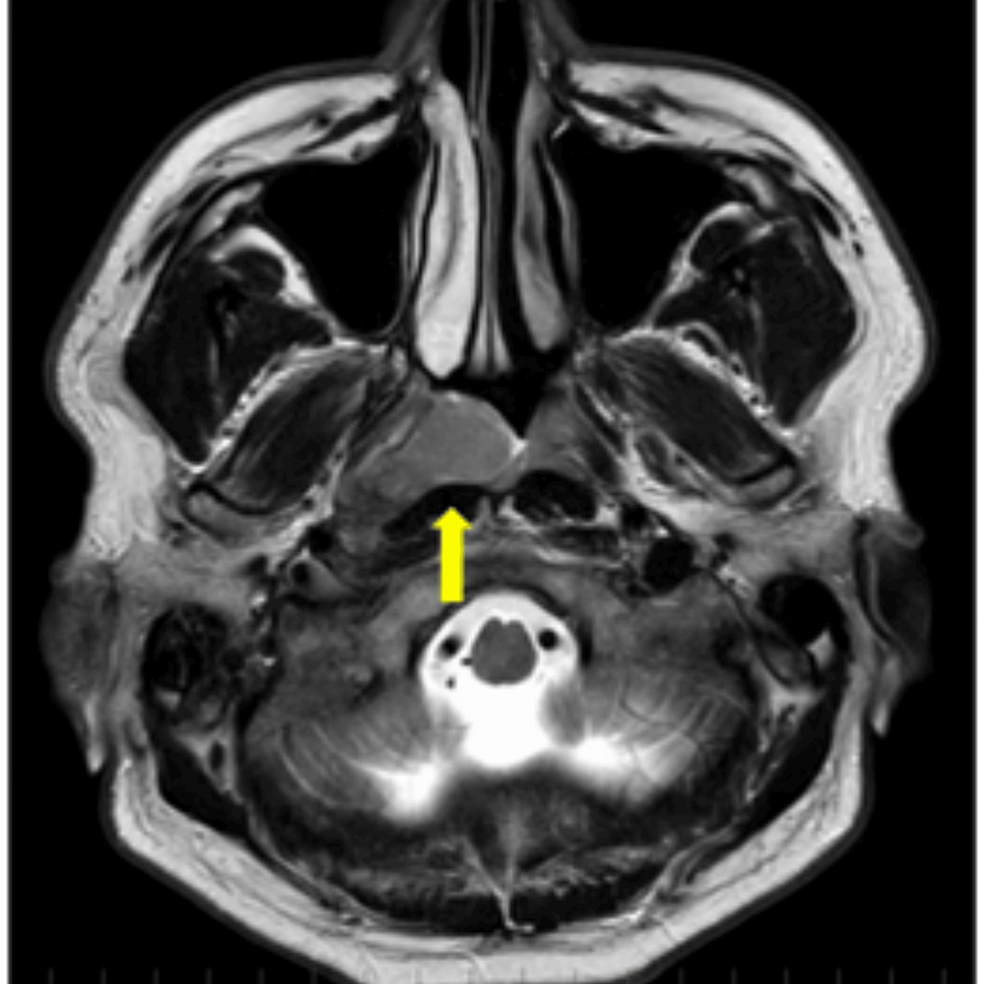
Magnetic resonance imaging at the initial diagnosis The tumor (arrow) extended into the parapharyngeal space and was diagnosed as cT2.

The second patient was a 47-year-old man. He was initially diagnosed with nasopharyngeal squamous cell carcinoma (cT2N1M1) (Figure 5). Distant metastases were detected in the liver and ileum. Multimodal therapy, including surgery, concurrent chemoradiotherapy, and systemic chemo-immunotherapy, was performed, and a complete response was achieved for all lesions. Despite continuing the immunotherapy, a locally recurrent lesion appeared 18 months after the start of treatment, and he was referred to our hospital. The tumor extended from the posterior wall to the left and right lateral wall. MRI showed a tumor thickness of 15 mm. The process up to photoimmunotherapy was similar to that used in Case 1. Cetuximab sarotalocan sodium was administered, with no adverse effects.

**Figure 6:**
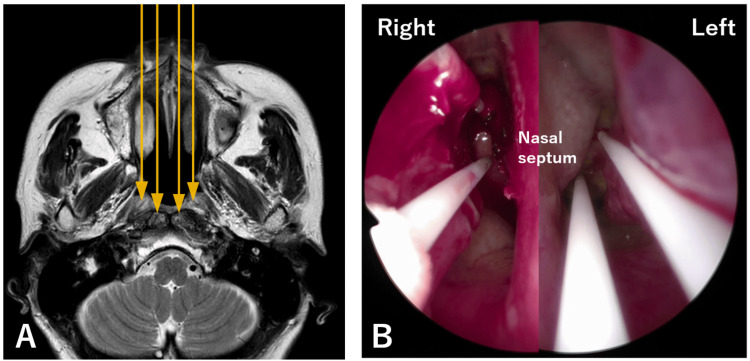
Schema and fiberscopic finding of the first photoimmunotherapy in Case 2 A: Schema of the first photoimmunotherapy using cylindrical diffusers. B: Fiberscopic findings in a composite image from both nasal cavities. A total of four cylindrical diffusers were used.

After induction of general anesthesia, adhesion between the nasal septum and the right inferior turbinate was recognized and resected. Two 100-mm needle catheters were used to puncture the posterior wall, one above the other, through each nasal cavity, for a total of four. Then, a 20-mm cylindrical diffuser was inserted into each catheter and illuminated (Figure 6).

**Figure 7:**
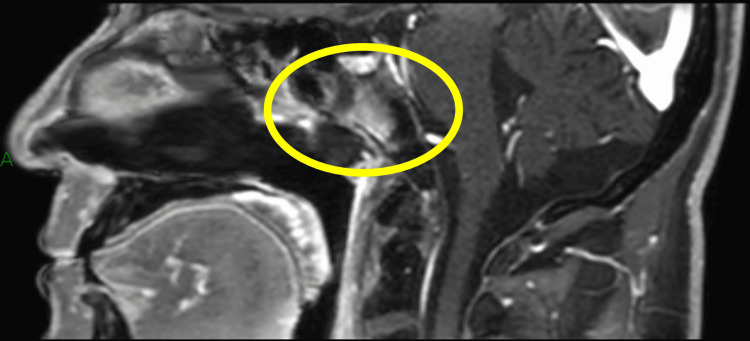
Magnetic resonance imaging one month after photoimmunotherapy One month after photoimmunotherapy, magnetic resonance imaging showed contrast enhancement at the clivus, which was suspected to be osteomyelitis.

He had a persistent headache after photoimmunotherapy. One month after photoimmunotherapy, an MRI showed that contrast enhancement was seen at the clivus, and osteomyelitis was suspected (Figure 7). However, it was cured in about a month without treatment. We determined that a residual tumor was present and planned a second photoimmunotherapy.

**Figure 8:**
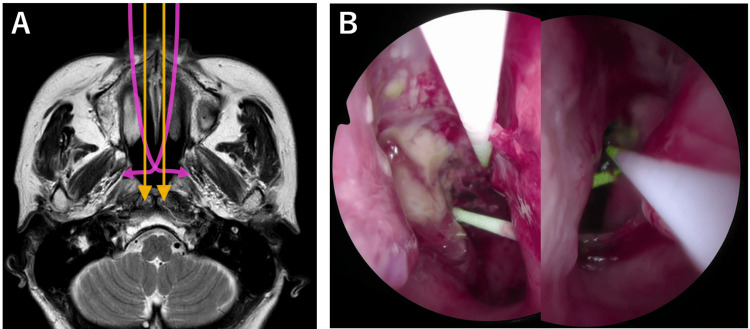
Schema and fiberscopic findings of the second photoimmunotherapy in Case 2 A: Schema of the second photoimmunotherapy in Case 2. B: Composite images from both nasal cavities at different times. A curved cylindrical diffuser punctured the area posterior to the pharyngeal orifice of the Eustachian tube through the contralateral nasal cavity.

A straight cylindrical diffuser was used to puncture the cephalic side of the lateral wall through one nasal cavity. A curved cylindrical diffuser illuminated the caudal side through the other nasal cavity. This was done again with the sides of the nasal cavities reversed (Figure 8).

One month later, an MRI showed tumor shrinkage. The residual tumor was mainly located on the left lateral wall. The third photoimmunotherapy was performed with the frontal diffuser illuminating the posterior wall and the cylindrical diffuser placed as in the second procedure. Subsequently, cervical lymphatic metastasis was found, and neck dissection was performed. Six months after the third photoimmunotherapy, local recurrence occurred, and the patient underwent a fourth photoimmunotherapy followed by systemic chemotherapy. The patient survived with cancer a year after the first photoimmunotherapy.

## Discussion

We successfully treated two cases of locally recurrent NPC with photoimmunotherapy. In the first case, a complete response was achieved and seven months have passed. In the second patient, local recurrence after multidisciplinary treatment was controlled as a stable disease for about one year. Such results could represent a breakthrough in the treatment of locally recurrent NPC after radiotherapy.

For residual or recurrent NPC, extensive surgery or re-irradiation has been considered [5]. Salvage endoscopic nasopharyngectomy is considered a good option for rT1 and rT2, and a relatively good prognosis can be expected [6]. When an open approach is used, complications such as trismus, palatal fistula, and osteoradionecrosis have been reported and quality of life is decreased after salvage nasopharyngectomy via the maxillary swing approach. So, salvage surgery for local treatment failure can be challenging, with potentially severe morbidities [6].

With re-irradiation using intensity-modulated radiotherapy (IMRT), the local control rate is 72% [7]. However, re-irradiation has the risk of severe toxicities such as mucosal necrosis, massive hemorrhage, and radiation encephalopathy [8]. The rates of >Grade 3 mucosal necrosis, hemorrhage (from the internal carotid artery or its branches as a result of mucosal necrosis), temporal lobe necrosis, cranial neuropathy, trismus, dysphagia, and hearing loss were observed in 34%, 19%, 19%, 14%, 20%, 11%, and 19%, respectively. It has been reported that the event rate for Grade 5 toxicities was 33% [7].

When neither salvage surgery nor IMRT is adapted, the drug therapy has been considered. Platinum-containing doublet chemotherapy regimens are generally considered the first-line systemic therapy for recurrent or metastatic NPC. It has been reported that the PFS was seven months with gemcitabine and cisplatin [9].

In comparison with the above modalities, photoimmunotherapy has several advantages. First, it is relatively minimally invasive. In our experiences, only grade 2 adverse events have occurred (pain, laryngeal edema, suspected osteomyelitis). The mechanism by which laryngeal edema occurred with this treatment is not clear. In the phase 1/2a study of photoimmunotherapy in locoregional recurrent head and neck cancer, which did not include NPC, laryngeal edema ≤Grade 2 was observed in two of 30 patients (6.7%) and there was no laryngeal edema ≥Grade 3. That study found that treatment-emergent adverse events ≥Grade 3 were observed in 63.3% of patients [3]. In all the cases we have treated, the length of hospital stay was four to five days after surgery. Furthermore, photoimmunotherapy can be repeated. In Japan, up to four repeat operations are covered by insurance.

The key to photoimmunotherapy in terms of technique is to irradiate the target lesion as accurately as possible. The choice of diffusers depends on the localization of the tumor. A frontal diffuser is used for superficial lesions, a cylindrical diffuser is used for deep lesions. An accurate approach to nasopharyngeal tumors is difficult due to their anatomic characteristics. The nasal cavity is a narrow operating space for photoimmunotherapy. In the second photoimmunotherapy Case 1, we tried to place a frontal diffuser 34 mm from the posterior lesion but could not do so because the frontal diffuser hit the posterior end of the nasal septum. Thus, we placed it 30 mm away and illuminated a diameter of 17 mm. In Case 2, an adhesion between the nasal septum and inferior turbinate due to past irradiation was recognized and required resection. So, in many cases, photoimmunotherapy will need to be preceded by surgery to widen the operating space, such as turbinate reduction and septoplasty for nasal septum deviation.

In the first photoimmunotherapy in Case 2, we punctured the cylindrical diffuser into the posterior wall directly through the nasal cavity. As a result, the lateral lesions remained. We think the illumination did not adequately reach the lateral wall. Therefore, we used a curved cylindrical diffuser to illuminate the lateral wall through the contralateral nasal cavity in the second procedure. We are now performing photoimmunotherapy with such technical improvements. In addition, future development of devices is expected such as side-fire (emission directly from the tip) and a short focal length frontal diffuser and cylindrical diffuser to illuminate inside bone lesions. Such devices could expand the indications of photoimmunotherapy and cure more patients. Since we observed favorable results in both cases; however, the long-term prognosis with photoimmunotherapy is still unclear, so further research is required.

## Conclusions

Local recurrence of nasopharyngeal carcinoma was previously difficult and the prognosis was poor. Systemic drug therapy was more often indicated. Within this background, good local control was achieved in our case.

Photoimmunotherapy may be an effective treatment for local recurrence of nasopharyngeal carcinoma after radiotherapy. On the other hand, this therapy is still new and there are many unknowns. Further data collection is needed.

## References

[1] Nakajima K, Takakura H, Shimizu Y, Ogawa M (2018). Changes in plasma membrane damage inducing cell death after treatment with near-infrared photoimmunotherapy. Cancer Sci.

[2] Ogawa M, Tomita Y, Nakamura Y (2017). Immunogenic cancer cell death selectively induced by near infrared photoimmunotherapy initiates host tumor immunity. Oncotarget.

[3] Cognetti DM, Johnson JM, Curry JM (2021). Phase 1/2a, open-label, multicenter study of RM-1929 photoimmunotherapy in patients with locoregional, recurrent head and neck squamous cell carcinoma. Head Neck.

[4] Kobayashi H, Choyke PL (2019). Near-infrared photoimmunotherapy of cancer. Acc Chem Res.

[5] Chen YP, Chan ATC, Le QT, Blanchard P, Sun Y, Ma J (2019). Nasopharyngeal carcinoma. Lancet.

[6] See A, Chu C, Kiong KL (2021). Surgical salvage of recurrent nasopharyngeal cancer- a multi-institutional review. Oral Oncol.

[7] Leong YH, Soon YY, Lee KM, Wong LC, Tham IW, Ho FC (2018). Long-term outcomes after reirradiation in nasopharyngeal carcinoma with intensity-modulated radiotherapy: a meta-analysis. Head Neck.

[8] Lee AW, Ng WT, Chan JY (2019). Management of locally recurrent nasopharyngeal carcinoma. Cancer Treat Rev.

[9] Zhang L, Huang Y, Hong S (2016). Gemcitabine plus cisplatin versus fluorouracil plus cisplatin in recurrent or metastatic nasopharyngeal carcinoma: a multicentre, randomised, open-label, phase 3 trial. Lancet.

